# AGEISM: AGING KNOWLEDGE AND PERSPECTIVES OF THE AGING NUTRITION NETWORK

**DOI:** 10.1093/geroni/igac059.2757

**Published:** 2022-12-20

**Authors:** Savannah Schultz, Sarah Francis, Alexandra Bauman

**Affiliations:** Iowa State University, Ames, Iowa, United States; Iowa State University, Ames, Iowa, United States; Iowa Department on Aging, Des Moines, Iowa, United States

## Abstract

Addressing ageism is essential to support older adult well-being. The Nutrition and Aging Resource Center (NRCNA) (1) examined the general characteristics, aging perceptions, and preferred training modalities of national aging nutrition network providers and (2) measured the impact an ageism webinar had on topic familiarity, knowledge, and behavioral intent. Frequencies were run to analyze the characteristics, aging perceptions, and behavioral intent while Wilcoxon signed rank tests measured outcome changes. Ageism was assessed via the W.H.O. Ageism Quiz (AQ) while aging perceptions were measured with the Facts on Aging Quiz (FAQ) and Expectations Regarding Aging (ERA). Respondents (n=1910) were primarily female (63%), non-Hispanic (90%), and white (87%) with an average age of 50 years. At least one-half had moderate ageism and aging perspective scores [Mean scores: AQ = 4.9±1.9 (max 8); FAQ =5.9±1.8 (max 10); ERA = 54.4±18.7 (max 100)]. The most preferred continuing education format was live webinars (44.3%). In response, a one-hour webinar about unconscious aging bias was offered in June 2022. A retrospective evaluation was used to assess the webinar impact (n=130 responses). A significant increase in subject matter familiarity (Z= -8.8, p < 0.0001) and knowledge (Z= -8.5, p < 0.0001) was noted. Further, analyses revealed positive attitudes (beneficial, good idea), perceived behavior control, indirect social norms (client support), and intent to reflect on past interactions with an older adult(s) to see how age-related biases might have influenced those interactions. These results illustrate the need for and the impact of ageism awareness training among those who work with the aging population.

